# Outcome-Focused Dance Movement Therapy Assessment Enhanced by iPad App MARA

**DOI:** 10.3389/fpsyg.2018.02067

**Published:** 2018-10-29

**Authors:** Kim F. Dunphy, Tessa Hens

**Affiliations:** ^1^Mackenzie Post-Doctoral Research Fellow, Creative Arts Therapy Research Unit, University of Melbourne, Melbourne, VIC, Australia; ^2^Dance Movement Therapist, Bayley House, Melbourne, VIC, Australia

**Keywords:** assessment, dance movement therapy, intellectual disability, National Disability Insurance Scheme, iPad app, eco-systematic assessment, person-centered

## Abstract

Healthcare and human services are increasingly required to demonstrate effectiveness and efficiency of their programs, with assessment and evaluation processes more regularly part of activity cycles. New approaches to service delivery, such as the National Disability Insurance Scheme (NDIS) scheme in Australia, require outcome-focused reporting that is responsive to the perspectives of clients. Eco-systematic approaches to service delivery and assessment consider the client as part of an interconnected web of stakeholders who all have responsibility for and contribute to their development and progress. These imperatives provide challenges for modalities for which there are not well-established assessment approaches. Dance movement therapists face particular difficulties in this respect, as they have few assessment tools that are practical for regular use. Existing dance movement therapy (DMT) assessment approaches largely do not yet prioritize input from clients. This article addresses these challenges in reporting a trial of iPad app *MARA* (Movement Assessment and Reporting App) developed for assessment in DMT. *MARA* is applied in a program for adults with intellectual disability (ID) over 16 weeks. Assessment data is gathered utilizing the app's features: two researcher-therapists undertake quantitative scoring that *MARA* aggregates into graphs, substantiated by qualitative note-taking, photos, and videos; and clients provide feedback about their progress stimulated by viewing photos and videos. A sample graph generated by *MARA* and supporting notes and a report drawn from data are provided. Responses to reports from program stakeholders (12 participants, 12 families, 11 center staff) gathered through interviews and focus groups are discussed, and researcher–therapists' reflections are detailed. The benefits of using *MARA* reported by researcher–therapists include strengthened capacity to focus on participant outcomes, assess efficiently, plan and make decisions for the program, and communicate participants' progress to stakeholders. Family members perceive reports drawn from data gathered in *MARA* to be useful in enabling better understanding of the DMT program and participant outcomes and potentially to support NDIS service planning. Managers perceive the potential value of data in these reports for quality control and resource decisions, while other staff confirm the therapists' perspective that reports offer the possibility of improved communication and collaboration between center staff.

## Introduction

This article reports the trial of an iPad app *MARA* (Movement Assessment and Reporting App; Dunphy, 2018a) to assess the progress of clients with intellectual disability (ID) in a dance movement therapy (DMT) program. The research task was to discover whether *MARA* can support dance movement (DM) therapists to undertake regular assessment that informs their practice, include the perspectives of participants, and provide meaningful information for participants, their families, and other stakeholders. *MARA* was utilized in DMT program led by author Hens at Bayley House (BH), a day center for clients with ID in Melbourne, Australia. The center is currently involved in significant changes to service provision in response to requirements of the new National Disability Insurance Scheme (NDIS) for outcome-focused practice that responds to clients' choices for their own lives.

*MARA* was developed as a practical tool to support assessment by DM therapists, underpinned by an *Outcomes Framework for Dance Movement Therapy* (Dunphy and Mullane, 2018). A previous research project explored the application of an earlier version of the app (Dunphy et al., 2016). Findings indicated its potential to support DMT assessment, along with some suggestions for additional technical elements, notably security features. Further research to explore client contributions to assessment was also recommended.

The current project trials the next version of the app, improved in response to those earlier findings. Assessment options enabled by *MARA* comprise quantitative scoring, qualitative note-taking, photos, and videos. Two researchers, authors Dunphy and Hens, both trained DM therapists, used all of these to assess participants' progress. This data generated was used to stimulate participants' reflections on their own experience in the DMT program. Then data generated through the app and participant feedback was integrated into progress reports and distributed to stakeholders (participants, families, and center staff). These stakeholders then offered responses to the reports in interviews and focus groups.

This article focuses on *how* the assessment was undertaken, particularly the practical and technological aspects of the assessment process, rather than *what* was assessed. A detailed examination of the content, measures and scales of the *Outcomes Framework* and the actual data generated in the assessment process is being prepared for publication in a separate article.

The article begins with literature review of contemporary imperatives in health and human services including evidence-based, outcome focused practice, person-centered approaches, and prioritization of client self-advocacy and voice, and their implications for DMT assessment. Issues for assessment of outcomes with people with ID in DMT are discussed. Then the research method is outlined, both the gathering of data about the app's use and stakeholders' responses. The process of creating reports from data generated by *MARA* is briefly outlined. Findings discussed include analysis of stakeholders' responses to reports and therapists' experience using *MARA* in the DMT sessions and reflections on the diverse data options utilized. The article concludes with considerations for future development of *MARA* and recommendations for research to advance meaningful and practical outcomes-reporting in DMT.

## Background

Health and human services internationally are increasingly required to demonstrate effectiveness of their programs, and to operate within evidence-based paradigms (Laska et al., 2014; Melnyk et al., 2014). Evaluation processes are more regularly expected as part of activity cycles (Ashton, 2015; Elsayad et al., 2015) and more frequently underpinned by outcomes frameworks (see for example, Lamb et al., 2015; State of Victoria Department of Health Human Services, 2016). Person-centered approaches are becoming more central in these fields, in contrast to earlier problem- or diagnosis-focused models prevalent in medical disciplines (Raskin et al., 2008; Brooker and Latham, 2016).

These changes are occurring alongside significant re-orientation of service provision for people with a disability toward client-led choice making and self-advocacy. The United Nations Convention of the Rights of Persons with Disabilities (United Nations Division for Social Policy and Development, 2008) places those with disabilities and their families and carers at the center of decision-making related to life goals and service provision (Ottman and Crosbie, 2013).

In Australia, these changes are reflected in the establishment of the NDIS in 2013. The initiative is underpinned by a philosophy of individual empowerment and choice to enable people with disability to enjoy an “ordinary life” (National Disability Insurance Agency, 2017). This includes increased access to services that facilitate meaningful and inclusive social participation (National Disability Insurance Agency, 2017).

The NDIS has catalyzed a major restructure of services to Australians who experience disability, replacing block-funding to providers with individualized funding packages controlled by service users. Service providers are impelled to undertake outcome assessment and reporting with more rigor than previously, including a greater focus on participants' perspectives.

This transformation of the socio-political landscape and imperatives from person-centered approaches challenge the notion of people with disabilities as being passive recipients of care or treatment. Policy makers and service providers are obliged to foster greater self-determination in those experiencing disability, including valuing of their experiential expertise at least as highly as professional “experts” (Thill, 2015). This increases the need for robust methodologies for self-assessment by service users (Thill, 2015).

However, obtaining input of clients with ID about their own experience can be difficult, even with best intentions. Barriers to indicate choice and reflect on progress include challenges with communication, recall, generalization, and abstract thinking (Ottman and Crosbie, 2013). Many people with disability also experience low societal expectations around their ability to self-advocate, hindering confidence, and capacity to share opinions (Thill, 2015). However, the skills people with ID need to self-advocate can be developed, through strategies including additional time, practice, and consideration of communication preferences (Thill, 2015). The importance of honoring non-verbal communication such as movement and vocalization in therapeutic exchange and disability service delivery is noted by Edwards (2017).

Methodological approaches that have been effective in capturing the experiences and views of people with ID include mixed-method discussions and interviews, especially if they are structured with focusing questions and interviewer prompts. Additional assistance can be provided by visual or augmentative communication supports, observation, and input from sensitive carers (Ottman and Crosbie, 2013).

An eco-systematic approach to assessment is recommended for clients with ID (Hoo, 2017). This approach views therapeutic interventions as being significantly influenced by interactions between individuals and their social networks. Because of the communication challenges and other barriers people with ID face, meaningful goal setting, and responsive assessment requires effective collaboration between therapists, participants, their families, carers, and other stakeholders. The eco-systematic approach also recognizes the importance of transferability of learning and skills from therapeutic interventions (Hoo, 2017).

DM therapists face particular difficulties in responding to all of these demands. They are challenged to provide evidence-based assessment of their programs (Karkou, 2010; Caldwell, 2013; Cruz, 2013; Dunphy et al., 2016 for reasons including lack of assessment frameworks that: are user-friendly and comprehensive (Powell, 2008; Cruz and Koch, 2012); adequately describe observable movement (Powell, 2008); and accessible for therapists without extensive specialized training (Koch et al., 2001; Cruz and Koch, 2012). Existing assessment models often do not consider participant perspective as part of systematized approaches, nor provide data that is meaningful for therapists as well as other stakeholders, including clients themselves (Dunphy et al., 2016).

DM therapists are required to assess effectively while actively moving throughout therapeutic processes, often working with groups of clients simultaneously. They are frequently employed as sole practitioners of their discipline, resulting in some degree of professional isolation, and under-supported professional practice. Assessment tools and outcomes relevant for their speciality may not be aligned with broader assessment frameworks used in workplaces (Dunphy et al., 2016). DM therapists' preferences for informal assessment systems and measures rather than more systematic approaches (Powell, 2008; American Dance Therapy Association, 2017) is also likely to be contributory in these challenges (Meekums, 2010).

However, our view of the most challenging issue for robust assessment in DMT, arrived at after extensive consultation with DM therapists across the world during the development of app *MARA* (Dunphy, 2018b) is the lack of impetus for robust reporting from agencies employing them. Without a demand from employers, not surprisingly, DM therapists are less compelled to invest in time-consuming assessment processes. This lack of impetus from others can be compounded by ambivalence from some practitioners about the desirability of an outcome focus in their work (Meekums, 2014).

An earlier scan of the literature indicated modest documented developments of technological tools including apps being employed in organized care settings and in creative arts therapy practice (Dunphy et al., 2016). However, many of these tools are utilized for basic admin functions rather than to inform clinical decision-making. Other apps are used to facilitate therapeutic processes to be assessed (e.g., Choe, 2014; Mattson, 2015) rather than providing an assessment system and reporting possibilities.

A previous international survey of DM therapists found no use of technological tools to support assessment (Dunphy et al., 2016). These findings were supported by our discussions with DM therapists across several countries in the intervening years. A scan of the main international journals over their histories (*American Journal of Dance Therapy, Body Movement and Psychotherapy*, and *The Arts in Psychotherapy*) supported this finding, with no documentation of technology used for assessment in DMT yet published.

## Methods

### Assessment tool MARA

The iPad app *MARA* was developed to provide a practical option for quality assessment by DM therapists. *MARA* is underpinned by an *Outcomes Framework for Dance Movement Therapy* created specifically for this task, with outcomes structured across five domains: physical, intrapersonal/emotional, cultural, cognitive, and interpersonal (Dunphy and Mullane, 2018) (overview attached as Appendix 1). The provision of an outcomes framework that includes specific measures across a comprehensive set of domains is posited to catalyze the DM therapist to first clearly identify outcomes of the therapeutic process that s/he and the client have agreed to work toward, and then to focus intently on assessing the client's progress toward these, selecting a score for each instance of a movement behavior observed. Previous users report their observation practices being “sharpened” in using *MARA*, especially in its requirement that a numerical score for client's access to each objective be selected (Dunphy et al., 2016).

This *Framework* is informed by a person-centered (Brooker and Latham, 2016) and strength-based approach, intended to support therapists to make judgements that are considerate of individuals' strengths and lived experiences. Assessment scales are not norm-referenced but referenced against the therapist's judgement of the participant's current capacity on each objective. The therapist scores how close s/he believes the participant to be in achieving their current potential, with a high score indicating observed actualization of skills and capacity, and a low score indicating observed potential as yet under-developed or enacted. This offers the possibility for the scale to be adjusted over time in response to changes in participants' capacity (improved or otherwise) as a result of the therapeutic process or other factors.

The app's facility to record and export assessment data in multiple formats (numerical graphs, written notes, photos, and videos) and save it in a client's profile filed by session, time, and date is intended to enable evidence-based assessment, with therapists' scores and written observations triangulated by these multi-media forms. Quick scoring with taps on *MARA* is designed to facilitate more regular and efficient assessment than is possible with paper-based processes that need additional post-session work to process into reports.

The facilitation of improved assessment processes is expected to enable more robust evidence of client progress (or lack of) over time, and therefore support more accurate reflection on what is working, or not, in DMT practice. *MARA*'s security features including two levels of password protection enable data to be secure as it is gathered and stored on the app.

In addition to these hypotheses about *MARA*'s potential for assessment for DMT programs broadly, *MARA* was considered to be appropriate for BH's DMT program because of its alignment with person-centered principles. Its potential usefulness to support an “eco-systematic” approach to assessment (Hoo, 2017) was noted, given its capacity to capture assessment data in various formats from therapists and clients and to meet differing needs of program stakeholders. In this project, we explored all of these claims of *MARA* app.

### Research participants

Research participants were:

- authors, researcher Dunphy, and researcher-DM therapist Hens, acted also as participants trialing and reflecting on experiences using *MARA*;
- program participants (12), who all had ID, defined as an IQ score of 70 or below, arrested brain development or brain injury resulting in lifelong impairment of cognitive, motor, language, and social skills (Australian Institute of Health and Welfare, 1997). Several participants use non-verbal communication solely or primarily;
- center staff (11), three BH managers with responsibilities for center-wide planning and reporting, and eight keyworkers with responsibilities for planning for individual clients;
- parents of program participants (12), who had a major caring role for each participant.

### The dance movement therapy program

The DMT program in which *MARA* was trialed was a new initiative for BH and DM therapist Hens. This project had the additional function of supporting Hens to develop a workable assessment process. The program was underpinned by the recognition of the potential of DMT to increase wellbeing through emotional, physical, and cognitive integration (Levy, 2005). Objectives included increased physical mobility; development of emotional regulation/intelligence; fostering of creative expression and confidence; and expansion of social and communication skills. Clients self-selected and self-funded their participation.

Prior to participants' commencement in the program, the DM therapist undertook preparatory work to develop appropriate objectives. This included examination of clients' individual support plans (PCPs), consultation with participants, parents, and staff members regarding their goals for participation and support needs. Information for each participant including PCP goals, family details, key worker at BH, medical, and other information was saved into the appropriate section in *MARA* app. Then time was spent observing clients in the DMT session to confirm decisions re objectives to work on.

### Research design

The project had three phases: (1) Data gathering using *MARA*—researchers' quantitative scoring and qualitative note-taking, photos and videos; and clients' reflections on their own progress elicited in interviews, stimulated by photos and videos; (2) Reporting—creation of reports for clients and other stakeholders using data generated as above; and stakeholders' responses to these, gathered through phone interviews or written survey; (3) Researchers' reflection—reflective discussions between the researchers on the process of using MARA, the data generated, responses from stakeholders re reports, and the impact of all this on assessment and therapeutic practice.

#### Data gathering

**Figure 1:**
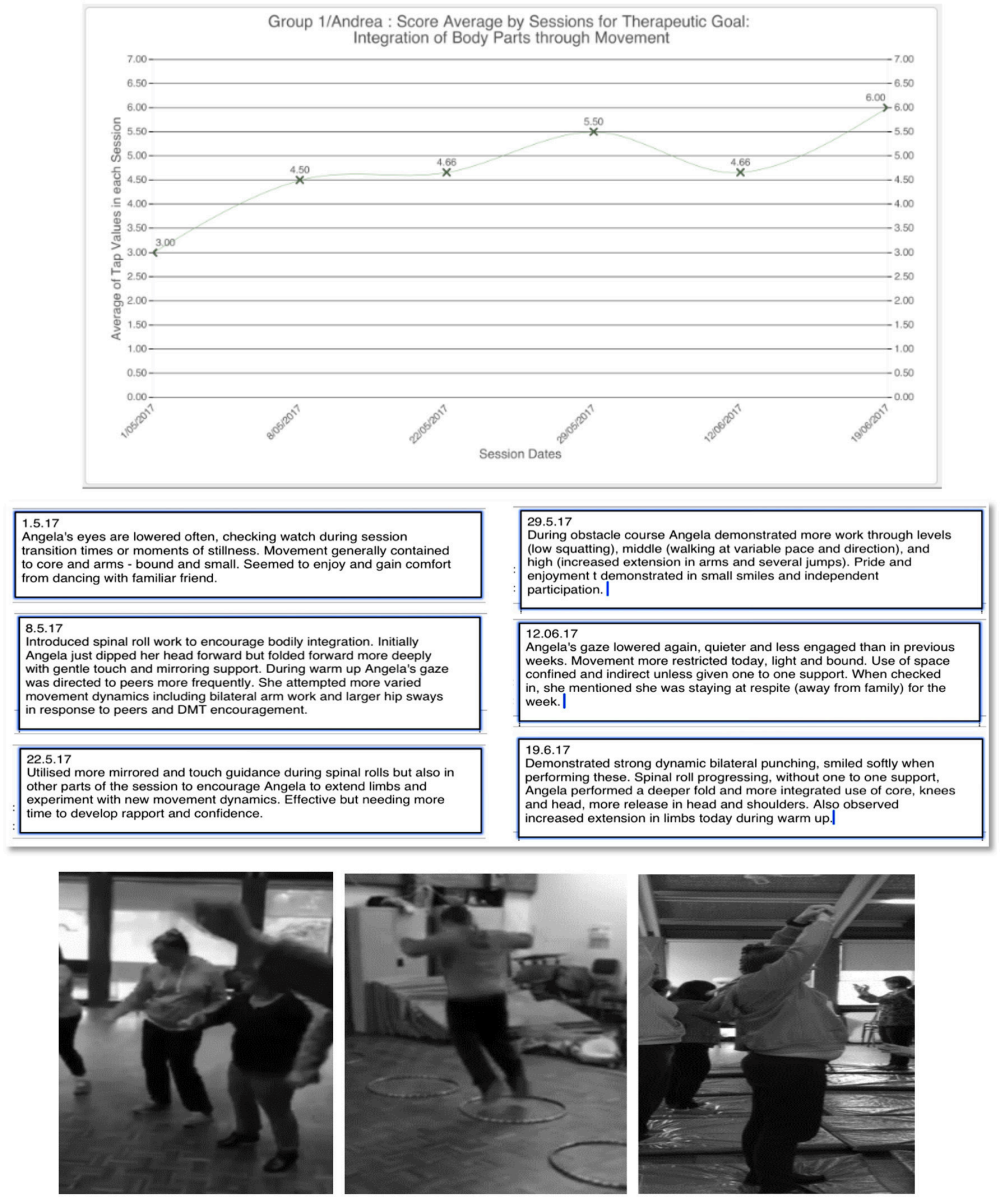
Graph, written note, and photos provided by *MARA* for Angela's score on Objective 1: Integration of body parts. Photo published with informed consent of participants.

Over 16 weekly sessions, researcher-DM therapist Hens delivered the DMT program and assessed clients, supported by non-DMT trained support staff. Dunphy contributed as additional assessor. Both researchers used *MARA* to assess participants' progress toward two objectives: 1. *Physical domain: Body organization and connectivity—Integration of body parts*; and 2. *Interpersonal domain: Connection with others—Appropriate give and take in relationship*. Assessment was undertaken in the first two sessions as base-line; two sessions mid-way; and in the final two sessions. Quantitative ratings were made by the therapists tapping the app to record their score for each objective. Each participant was scored on each objective at least once per session. Dunphy scored during each session as many times as she saw a movement or action relevant to the objective, while Hens scored immediately after the session, making as many scores as she could remember for each moment relevant to the objective. *MARA* averaged scores for each participant on each objective per session and formatted these into graphs. This numerical data was substantiated by qualitative notes for each objective, and photos and video clips taken throughout sessions (see example in Figure 1).

Program participants were invited to reflect on their experiences in DMT sessions in interviews with one researcher. These occurred during the DMT session time toward the end of the 16-week period. Participants were interviewed individually, outside the DMT space to minimize distractions, ensure confidentiality, and to enable support for individual communication needs. The therapist first explained program objectives, then each participant viewed photos and short videos of themselves taken during sessions. Participants were then invited to comment on their experience in the program and of seeing themselves, and their sense of progress toward objectives. Verbal communication was augmented with symbols and visual aids that participants were experienced in using, where appropriate. Non-verbal responses including movement, vocalization, facial expression, and gesture were also recorded. This data was uploaded into each participant's file in MARA.

#### Reporting

Reports were developed for each participant utilizing data gathered and saved into *MARA*. These included scores and notes made by therapist Hens, participants' responses and supporting photos and videos, all summarized and interpreted into plain language. These reports were distributed to participants, their families, and center staff by email or hard copy.

Then, responses to these assessment reports were investigated. Participants' family members were interviewed in person or by phone, center managers were interviewed, and keyworkers were interviewed or contributed in a focus group (Sample questionnaires attached as Appendices 2 and 3). Responses to reports were invited, considering their readability and length, insight provided and usefulness of the data in the documents for current or future planning or reporting needs. These responses were analyzed and presented in summary form.

#### Researchers' reflections

The researchers undertook reflection discussion on the use of MARA for assessing and reporting, after each assessment session and at the end of the period. Topics addressed were: the technical aspects of assessing using MARA; decisions we made about when in the session, and in the series of sessions to assess and how often; and data generated about participants on each objective, in numerical scoring, notes, photo, and videos. Then we reflected on the overall experience, both the assessment and reporting processes and responses to all of it from stakeholders.

We followed informal discussion of comparison of our scores with estimates of interrater reliability based on Intra-class correlation coefficients (ICC). These were based on assessment of 12 participants on two different occasions by both researcher-therapists, on measures *Body organization and connectivity* and *Connection with others*. Type of agreement assessed was *absolute agreement*. ICC measure for single rater was used, to a 95% confidence interval.

### Ethics procedures

Ethics approval was gained through University of Melbourne Human Research Ethics Committee (ID: 1647380). All processes required by the Committee were undertaken, including obtaining informed consent from participants. BH's regular consent processes were followed, given participants' cognitive impairments, with participants' elected advocates (all family members) supporting participants to consider issues of consent and signing the consent form on their behalf. Specific informed consent about use of photos was also obtained from appropriate participants, with participant “Angela” whose report and photo appears, supported by her advocate to provide consent for use of her first name as well. Augmenting this were discussions with participants and monitoring of their responses before and during sessions to ensure that they were comfortable with the researchers' processes, including use of MARA.

Information about the researchers' privacy and confidentiality processes was shared with participants in Plain Language Statements. Data was stored safely in locked facilities at the Creative Arts Therapies Research Unit at the University of Melbourne, or on the researchers' password protected laptops and iPads. Center staff and participants were kept informed about the research process through occasional newsletter items.

The authors advise of their dual roles in this project. Researcher Dunphy is also the inventor of app MARA and Hens is a staff member of BH leading their DMT program. While both researchers intended to bracket out vested interests in *MARA* and the program, respectively, from our research, we nevertheless acknowledge these as potential influences.

## Results

### Reflections on assessment process

This section reports researcher-therapists' experiences of using *MARA* for assessment elicited in reflective discussions, firstly with respect to program and session planning. Hens commented on the impetus provided by *MARA* to clarify objectives for the DMT program, with the provision of outcomes, and associated measures helpful in supporting her to identify objectives to assess against. She found that outcomes and associated measures in *MARA* that were suitable for her DMT program could also be adequately aligned with participants' existing PCP goals and new outcome measures required by NDIS. For example, a DMT program objective measured by *MARA* as “give and take in social interaction” was aligned with a participant's PCP goal of “building confidence and the ability to speak out,” and the NDIS' overarching outcome of “social inclusion.”

She experienced the use of *MARA* beneficial in decisions about the appropriate number of outcomes to assess. An initial intention to assess each client on different outcomes because of their different needs and PCP goals proved too challenging, given the practical limits of a session involving 12 clients. She then made the decision to assess the same objectives for all participants, choosing two that were appropriate for all group members. She found that measuring only two objectives for each participant was manageable as an assessment task and provided sufficient data for monitoring and reporting of participants' progress.

Both researchers concurred that the decision to assess in six sessions across the 16-week period seemed satisfactory. Assessing clients for a first, baseline, measure on two objectives seemed better undertaken across two sessions rather than one, given that both clients and therapists were engaging with new material. As assessors, we sometimes found it difficult to “see” anything to assess in the first session, as clients familiarized themselves with new movement material. Assessing in the two middle sessions for the period was experienced as useful, providing a focal point for considering participants' progress across that period and providing a focal point to consider adjustment of the program if needed to better support clients' development. The decision to assess across the final two sessions also seemed appropriate, enabling the therapists to make a thorough assessment of progress across the program, and reducing any anomalies that might have impacted scores for a single session.

Figure 1 depicts a graph created by *MARA* from therapists' scores for client Angela on Objective 1, *Integration of body parts* substantiated by qualitative notes and illustrated by photos. The graph indicates a gradual positive change over the period, from average score of three to six by the final session.

Both researchers considered our decision to score each client on both objectives every session a prompt for rigorous thinking, especially as each session was followed with reflective discussion. The process of numerical scoring seemed useful for several reasons. It focused our discussions specifically around stated objectives and what we had “seen” of each client in relation to these each session and provided direction for future session planning. For example, the dip in the score for client Angela in the fifth assessed session after weeks of steady positive progress, as depicted above, stimulated a conversation about factors that might have been causal in that change, and strategies the DM therapist might use to address this in future sessions. An additional benefit was that this discussion acted as a catalyst for supervision, with Hens' developing skills as a DM therapist supported or challenged by a perspective from longer-experienced colleague Dunphy.

We were interested to find that scores we had made independently of each other (Dunphy during the session and Hens when the session was over) were most often within one point of each other's. In instances where our scores were widely divergent, we discovered that this was often because we had observed different moments in the session. With twelve or more people moving in the room at one time it was not possible to observe everything. Our justification to each other about what we had seen to lead to our scoring decisions prompted interesting discussion and a sense of sharpening of our “minds' eyes.”

This reflective discussion about the scoring process was followed by a structured inter-rater reliability estimate based on Intra-class correlation coefficients (ICC), reporting absolute agreement for average of raters. These results indicate good inter-rater reliability (Koo and Li, 2016) for Measure 1: Body connectivity and moderate for Measure 2: Social Connection.

Table 1 estimates of interrater reliability based on ICC.

**Table 1:** Estimates of interrater reliability based on Intra-class correlation coefficients (ICC).

**Measure**	**ICC**	**Estimate**	**95% Confidence interval**
1.Body connectivity	Single rater	0.80	0.59, 0.91
2.Social connection	Single rater	0.64	0.30, 0.84

### Program participants' responses

This section reports program participants' reflections on their experiences and progress in the DMT program. All twelve participants agreed to contribute to this stage of the research as well. This data was gathered through short individual interviews, prompted by verbal information about the objectives of the program and videos and photos of their participation. Most participants were able to share reflections about the DMT program, using verbal or non-verbal communication. This included comments about enjoyment of a particular dance sequence or activity, or description of feelings linked to experiences. However, eliciting responses about progress toward objectives proved challenging. We reflected that this may have been because of the cognitive processing required, and communication challenges we had anticipated, possibly exacerbated by participants having to leave the session and go to a separate room for an interview, and therefore being removed from the context and experiences the assessment was focused on. Participants may not have had much previous experience reflecting on progress toward objectives.

There was also an unexpected response, with one participant having a negative experience in seeing a video clip of herself in movement. We had anticipated that participants would enjoy the opportunity of supported reflection on their movement experiences, but this participant's response indicated that we needed to be more sensitive in future to ensure that this process of self-assessment was a beneficial learning experience.

### Reporting

At the end of the 16 weeks, a report for each participant was developed utilizing data gathered through the app, comprising therapists' judgements, and participants' responses. This began with an outline of the DMT program's overall goals linked to: the NDIS goals being absorbed into the center's planning processes; goals from participants' Person-Centered-Planning; and DMT program objectives for that period. Participants' progress toward objectives were reported, accompanied by two to three short illustrative videos or photos.

In considering what data to include in the report, we made the choice not to send data in graph form to clients and their families. We considered that reports including graphs might be disconcerting, given the very significant difference of this type of information from current more informal reporting processes used at BH. As well, the lifelong nature of ID and the challenges it provides means that progress, when it occurs, can often be very modest. We were concerned of the possibility that depictions of incremental progress might be most supportive for families and participants, and contrary to the program's strengths-based intention. Hence, we provided only qualitative comments based on the data and photos and videos to represent participant engagement and honoring of progress at whatever rate it occurred. These reports took the DM therapist about 45 min to prepare for each participant once the basic format was established.

#### Families' responses to reports

After the reports were distributed, the family member who was nominated advocate for each client was invited to share their response to these. All family members invited agreed to participate. Issues investigated were readability and appropriateness in content and length, provision of new information or insight about the DMT program or the client's participation and usefulness, particularly with respect to supporting applications to NDIS for funding for DMT involvement. Responses were mostly positive, with eight of the twelve family members finding the report “really easy” to read, and four finding it “quite easy.” Ten respondents agreed that the length and layout were appropriate, while two would have preferred less detail. Three parents perceived “significant” new insights, five reported “some” new insights, and four reported no new information about their family member. Those not encountering new information cited their family member's ability to communicate their experiences or their previous exposure to dance programs as reasons.

Eight family members described the information provided in the report as being “very useful,” while four found it “somewhat” useful. Those who found reports most useful were supporting participants who have communication challenges that restrict their ability to share experiences. For example, one parent reported that the videos assisted her to discuss the DMT program more directly with her son.

Family members indicated that information from reports helped them understand objectives of the program and therefore its potential contribution. Nine respondents commented on outcomes of DMT or skills developed in the DMT program being transferred to contexts outside it. This included enhanced mobility, improved emotional regulation including mood, relaxation, and communication skills, social skills, and confidence.

With respect to the potential usefulness of the report in applications to NDIS for funding of further DMT activity, six family members affirmed that they thought reports would be useful and six were unsure. A recurring explanation for this uncertainty was a lack of familiarity with the NDIS funding requirements.

#### Center staff: keyworkers

In one focus group and three individual interviews, keyworkers overall responded positively to the reports. One commented that this report was “one of the best assessment forms I have seen in this center.” Affirming comments included that the clear articulation of program objectives was useful, that reports were thorough, easy to understand and provided valuable information. The discussion about the reports seemed useful in itself, in its prompting of keyworkers' focus on the progress (or otherwise) of their client in areas possibly impacted by the DMT program. For example, one keyworker reported that her client had improved fine motor skills and balance, resulting in less accidents, during the time she had been attending DMT, which the worker attributed to her participation in the program. Keyworkers also commented on the potential benefits for participants in receiving positive affirmation of their own movement styles, through the report's strength-based emphasis.

These responses indicated the potential for such reports to be a conduit for collaborative work between DM therapists and other staff, because they helped the keyworkers see the relationship between activities in the DMT program and their broader roles supporting clients. However, there was also hesitation expressed, with keyworkers reflecting on the amount of work required to develop such detailed reports. They indicated the barriers for other staff, like themselves, if they were expected to create reports of this complexity, given their significant current responsibilities. This was particularly salient because reporting in this detail had not been part of their work requirements to date.

#### Center staff: managers

BH managers also had positive responses, with the most senior staff member interviewed commenting that a report she examined was “incredible.” The likely usefulness of such reports for agency management, given the impending requirement for outcomes-based reporting by NDIS, was confirmed, along with perceived value for information-sharing with families whose DMT participant had communication barriers. Potential benefits were also identified for clients, with one manager commenting that through the reports, clients could “see their own achievement and take pride in their work.” Another point made was the evidence provided by the report of DM therapist Hen's commitment and deep engagement with clients' needs. One manager commented that the report “shows that someone has been taking a keen interest in (each participant)…If everyone knew what was happening in this (DMT) program, they'd want to come.”

There were also hesitations about practicality of such a reporting process. Two of the three managers confirmed the keyworkers' concerns about the resource demands of preparing such reports. One manager wondered about the feasibility of a reporting process that expected would be time-consuming, in a workplace where this was not common practice and staff did not necessarily have the skills to do it. Other concerns raised were the accessibility of language and amount of detail that may be too much for keyworkers with significant caseloads of clients. More succinct information supported by more visual data was recommended.

## Discussion

### Usefulness of MARA for assessment

These results from a range of DMT program stakeholders confirm that the iPad app *MARA* offers potential for effective and practical DMT assessment. The process of assessing, along with data generated through *MARA* was experienced as valuable by we two researchers, in catalyzing focused planning and reflection. We found that 30 min of discussion and scoring was adequate to assess up to 12 clients, making the assessment process enabled by MARA accessible to DM therapists working in real world circumstances.

MARA's functions of video, photo, and notes containing participant feedback being saved and filed according to sessions, dates, and times proved useful. This automatic and systematic storing made this data easily accessible to share with participants and export for reports. The inter-rater reliability of researchers' scores was moderate to good, despite scoring being undertaken in less than ideal circumstances, with researchers undertaking the assessment process at different times (during and after the session) and not necessarily scoring the same moments.

Reports underpinned by this data were experienced as useful for program participants' families, keyworkers, and center managers. These findings support the potential for *MARA* to facilitate person-centered eco-systematic approaches to program delivery and assessment. However, it was also evident that reduction of detail and length of reports may be beneficial in maximizing stakeholders' engagement with them and increasing the possibility that the DM therapist could sustain such assessment practices within the real-world limitations of her sessional role.

In preparation for this trial, it became evident that it was not possible for DM therapist Hens to assess using the app at the same time as facilitating a DMT group, especially when supported only by non-DMT trained assistants. MARA's features such a tap-touch scoring and quick saving of photos and videos made assessment more efficient, but they did not eradicate the problem that DM therapists working with groups without skilled support must wait until the end of the session to record assessment notes or scores, and thus rely much on their memory. All previous assessment systems have also had this same limitation, but worse, given the amount of time required for note-writing by hand and other form. Thus, MARA does not completely eliminate issues low-resourced practitioners have had in assessing, but it reduces some of the challenges.

However, as discussed above, scores were similar between researchers, even with Dunphy assessing during the session and Hens immediately afterwards. This supports the possibility that adequate reporting can be enabled by *MARA* even if it does not happen right within the session. This makes it potentially suitable for the real world circumstances of most DM therapists, and an advancement from previous assessments tools because of its technological features.

### Considerations for future research

This trial only involved observations made by two DM therapists, both of whom had dual investment as researchers and with *MARA* or the DMT program. Only participants from one program, one population group, one setting, and one country contributed. Further trials of *MARA* with researchers and DM therapists who are less closely connected to its development, working with different population groups in other settings and locations, are required to substantiate the findings of this trial.

The current trial also included a modest exploration of client perspectives in DMT assessment. This was enabled by *MARA*'s note storage and multi-media options that enable participants to see and reflect on salient moments in the therapeutic session once the session is over. However, it was evident that much improvement could be made in responsive and inclusive methods for eliciting participant self-assessment for people with ID. Future considerations may include ways to invite participants' reflection while they are within the DMT space, to reduce the challenge of abstraction in responding when moved out of the space, and as a more sustainable option given limited staffing resources. One participant's negative experience suggested the need for skills in offering scaffolded information that would enable participants to focus on their growth and development (current or potential), rather than reinforcing deficiencies they may perceive in themselves.

Two other topics not explored in this study were the actual outcomes, measures, and scales used for assessment in MARA: their suitability for the task, comprehensiveness, and validity. This work is necessary for MARA to provide a tool of appropriate professional standard. Nor was the data about outcomes of participation in the BH DMT program considered. Both of these topics are being developed in separate articles concurrently.

Inter-rater reliability estimates were moderate to good in the uncontrolled environment of this study, with raters assessing at different times (during and after the session) and not necessarily the same moment of participants' engagement. Further testing, with raters assessing the same moments in participants' engagement in the same way (during or after the session), would enable better assessment of IRR for MARA and its underpinning Outcomes Framework.

### Recommendations for development of *MARA*

This trial of *MARA* indicated that its current quantitative, qualitative, and media options can support effective DMT assessment practice. However, the need for another data collection function was evident. While the therapist can score any objective any number of times in a session, these are currently formatted by MARA only into graphed averages per session. We noted that participants were often scored differently against the same objective at different times in a session and this range was not evidenced in the current graph format. Therefore, a further set of data we identified as potentially valuable to be enabled by MARA was variation of scores within a session. This would enable examination of moments when clients engage and advance toward their potential and other moments that are less successful.

We reflected on possible reasons for the different scores, identifying possibilities such as shifts in group dynamics, participants' familiarity, or interest in specific activities, and emotional or physical states that might fluctuate throughout the session. Data that underpinned this thoughtful reflection could enable the DM therapist to plan and facilitate sessions to maximize engagement and growth. This would also be a valuable topic for future exploration.

## Conclusion

This trial of the suitability of iPad App *MARA* for effective assessment in DMT practice indicates its potential, at least for client groups of people with ID. Reflections from two researchers, and interviews and focus group responses from 12 clients, 12 family members, and 11 center staff indicate that reports compiled with data generated through *MARA* were valuable to all stakeholders. The possibilities that *MARA* enhances for client perspective in assessment were evidenced, indicating both usefulness of the current format of *MARA*, and the need for strategies to enhance client engagement in this process.

The likelihood of a tool such as *MARA* to be useful in meeting agencies' needs for outcome reporting, stimulated by the new demands of the NDIS in Australia, was also supported. An additional feature for *MARA* was recommended, of graphs that provide information about client responses within sessions, to complement the current feature of data graphed across a series of sessions. Considerations for more succinct reporting that was less time consuming for the DM therapist to produce and stakeholders to read were salient, to ensure that evidence-based assessment could be a regular and valued part of DM therapists' practice.

## Author contributions

KD and TH contributed in all aspects of the study. KD led the writing and structuring of the article, while TH led the practical aspects of the study, including liaison with the research site and participants.

### Conflict of interest statement

KD is the developer and owner of iPad app MARA with colleague Sue Mullane. TH works as a contracted staff member leading the DMT program in Bayley House. The reviewer NG and handling Editor declared their shared affiliation.

## Supplementary material

Table S1 **Appendix 1**: Overview of Outcomes Framework for Dance Movement Therapy (Dunphy and Mullane, 2018). **Appendix 2**: Sample questionnaire for clients' parents/carers. **Appendix 3**: Sample questionnaire for managers and keyworkers. **Appendix 4**: Sample report for participant Angela.
