# Supplementary material for: Outcome-Focused Dance Movement Therapy Assessment Enhanced by iPad App MARA
Source: Front Psychol. 2018 Oct 29;9:2067. doi: 10.3389/fpsyg.2018.02067 (PMC6220569; doi:10.3389/fpsyg.2018.02067)
Supplement: Table S1 — Appendix 1: Overview of Outcomes Framework for Dance Movement Therapy (Dunphy and Mullane, 2018). Appendix 2: Sample questionnaire for clients' parents/carers. Appendix 3: Sample questionnaire for managers and keyworkers. Appendix 4: Sample report for participant Angela. [file Table_1.pdf]

Appendix 1: Overview of Outcomes Framework for Dance Movement Therapy (Dunphy & Mullane, 2018)

TOWARDS FLOURISHING FULFILLED INDIVIDUALS

DOMAIN	SUB-DOMAIN	THERAPEUTIC GOAL					
**Physical** *Stable, mobile, expressive and functional body*	**Body:** Organization and connectivity	Ability to access and utilize breath to support movement	Core-distal connection	Head-tail connection	Upper-lower connection	Body-half connection	Cross-lateral connection
	**Body:** Sequencing of movement through body	Simultaneous	Successive	Sequential	Body parts active-held		
	**Effort:** Access to movement qualities	**Space** (Direct – Indirect)	**Weight** Active (Light – Strong)	**Weight** Passive (Limp- Heavy)	**Time** (Sudden – Sustained)	**Flow** (Bound-Free)	
	**Shape:** Access to body shaping	Directional shaping	Space carving	Shape flow	Shape qualities		
	**Space:** Use of body in space	Access to kinaesphere (near, mid, far reach)	Access to planes	Spatial intention	Access to levels in space	Body boundaries in space	
	**Fitness and co-ordination**	Stamina	Strength	Flexibility	Balance		
	**Relaxation**	Relaxed stillness	Deep, slow, regulated breathing	Release of tension			
**Cultural** *Creative, aesthetic expressive self*	**Creativity and aesthetic sense**	Connection between body sensations, feelings, thoughts and imagination	Creativity inspired or expressed	Experience of aesthetic enrichment	Making an aesthetic decision		
	**Knowledge, diversity, heritage**	New knowledge, insights, skills attained	Appreciation of diversity of cultural expression	Sense of belonging to shared cultural heritage stimulated			
**Emotional** *Healthy, regulated emotions*	**Emotional expression and regulation**	Capacity to identify own feeling/ emotional states	Appropriate mood / affect	Regulation of emotional expression	Energy attuned appropriately to The activity	Expression of appropriate feelings, emotions	Capacity to manage frustration, challenges
	**Fun, pleasure, enjoyment**	Level of engagement and enthusiasm	Smiling	Sense of safety experienced	Body Ease	Verbal/vocal communication	Playfulness
	**Reality orientation**						
**Cognitive** *Active enquiring mind*	**Initiative**	Indication of preferences and choice-making	Independent initiation of action or activity	Leading, taking ownership of an activity	Confidence in expression of self	Capacity to reflect on and share experiences	Indication of preferences, choice-making
	**Memory**	Sense of (appropriate) anticipation evident	Recall of movement sequences:	Recall of themes/activities from previous sessions	Meaningful exploration of life memories		
	**Executive function**	Reflective capacity?	Transfer, organize thinking, make connection, identify patterns	Integration of past, present and future embodied self	Bodyful sense of a positive future	Theory of mind	
**Inter-personal** *Satisfying relationships*	**Relationship**	Capacity to identify feelings/ emotional states of others	Appropriate response to others' emotions	Comfort in proximity to others	Appropriate use of personal space	Emotional connections	Appropriate eye contact
		Appropriate social connection: response, initiation, sustainment, release	Appropriate physical connection	Appropriate give and take	Expressive vocal /verbal communication	Appropriate vocal /verbal communication	
	**Connection**	Focus on the activity	Energy attuned appropriately				

Appendix 2: Sample questionnaire for clients' parents/carers

Research project: Assessment for dance movement therapy across contexts and cultures: advancing trials of an iPad app

QUESTIONNAIRE FOR CLIENTS AND PARENTS/CARERS

1.How easy or difficult was it for you to understand the assessment information in this report about your family member's progress in the therapeutic dance program?

☐ really easy ☐ quite easy ☐ difficult

If you found it difficult to understand, was it that:
☐ too much information was provided ☐ not enough information was provided
☐ the terminology used was unfamiliar to me
☐ other - please explain……………………………………………………

2.Did the assessment information provide you with new information or insight about your family member's progress in the dance program?

☐ significant new information or insight ☐ some new information or insight
☐ no new information or insight

Can you offer any comment?

3.How useful was the assessment information provided for you?

☐ very useful ☐ somewhat useful ☐ not useful

Can you offer any comment?

4.Do you think this information could provide any support for your requests for NDIS funding?

5. Is there anything else you would like to know about your family member's progress in the dance movement therapy program that was not provided?

Thank you for your contribution, Dr Kim Dunphy
(Responsible Researcher) k.dunphy@unimelb.edu.au

Appendix 3: Sample questionnaire for managers and keyworkers

Research project: Assessment for dance movement therapy (DMT) across contexts and cultures: advancing trials of an iPad app

INTERVIEW AND FOCUS GROUP INSTRUMENT FOR PROFESSIONALS

1. **How readable did you find the assessment information provided in these reports?**

2. **How appropriate were these reports for informing participants, families/carers and other staff about the DMT program in terms of format, content, layout and length?**

3. **Did the assessment information provide you with any new information or insight about the dance movement therapy program or client/s' progress**?

4. **Data usefulness**
    - How could/does data generated through this process inform your work?
    - What insights might be generated that are new and not obtainable through other assessment processes?
    - What is your perspective about the usefulness and relevance of the data generated for other stakeholders including clients, families/carers and other professionals?
    - Including for use for NDIS funding requirements?

5. **Is there any other information you would like that was not provided?**

Thankyou for your contribution, Dr Kim Dunphy (Responsible Researcher)
k.dunphy@unimelb.edu.au

Appendix 4: Sample report for participant Angela

Dance Movement Therapy Program Participant Report

Provided by Tessa Hens, dance movement therapist, Bayley House

Participant name: Angela X. **Term:** 2, 2017.

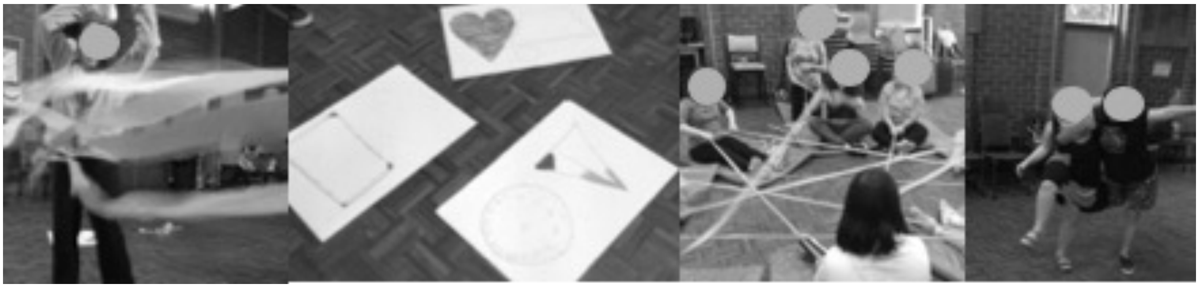

Figure 4: The dance movement therapy program. Informed consent provided by participants for use of photos.

This report is provided to support communication between our participants, their families and support staff to enrich participants' experience in the program. It includes information about the dance movement therapy program and its relationship to participants' NDIS and PCP goals. The final section offers the dance movement therapist's input about Angela's participation, and her own comments.

About the program

The dance movement therapy (DMT) program at Bayley House fosters the use of dance to address therapeutic goals meaningful to participants. It encourages enjoyment of all bodies' abilities to move and express through dance. The dance group aims to offers an accepting and safe space for participants to express themselves creatively and to support each other. It encourages self-awareness and ownership of each person's contributions and development. The program is informed by dance movement therapy and neuroscientific research which support the potential for dance and movement to enhance physical and mental health, emotional regulation, cognitive functioning and social skills.

Dance movement therapy sessions: what happens?

Each session comprises routine elements to support learning and personal development and to provide predictability and a sense of safety, including:

1. A greeting that incorporates symbolic movement and talk linked to participants' experiences or feelings for the day.
2. A warm up which isolates body parts and involves participants contributing a movement to the group.
3. Creative movement experiences to support objectives such as development of a wider range of movement dynamics, or social attunement skills.
4. Cool downs and stretches focussed on body awareness and integration, breath awareness and self-regulation.
5. Relaxation, experiencing stillness and rest to close the session.

Music is important part in the dance experience and is chosen to reflect the interests of the participants and to expose them to new genres and styles. Props are used to extend movement experiences and to support participants to recognise and connect with movement dynamics and conceptual themes. Music, props and variations in dance activities are designed to stimulate participants' interest and stretch cognitive and physical capabilities.

The relationship between dance and other areas of life

It is the intention of this dance program that the physical, expressive, emotional and cognitive gains participants may attain can transfer into other areas of their lives. For example, the ability to negotiate space more skilfully may enhance independence in daily life; while the ability to relax and release through practiced breath and stretching may provide tools for emotional self-regulation.

NDIS Outcomes, PCP Goals and Dance movement therapy objectives

NDIS Outcomes

NDIS outcomes relevant to this program are: *Independence, Social Inclusion, Health and Wellbeing.*

The DMT program supports progress towards those outcomes through:
-Independence: daily living skills are supported through increased physical health, strength and coordination
-Social inclusion: development of social skills through shared dance experiences in a safe and supportive group setting.
- Health and Wellbeing: physical and mental health are addressed through activities developing physical fitness and strength, and the ability to self-regulate through breath and movement work.

PCP goals

Angela's PCP Goals relevant to this program are: *To build confidence and the ability to speak out.*

The DMT program supports Angela's development of confidence and ability to speak out through strong emphasis on development of social skills and positive connections with others. By honouring both verbal and non-verbal communication, this program provides Angela with a range of communication tools to achieve positive connection and communication with others.

In dance movement therapy, Angela will have many opportunities to:
- share news and greet others with verbal and movement-based communication;
- work on creative movement activities in pairs and teams;
- express herself creatively in a safe and supportive environment alongside her peers.

Dance movement therapy program objectives

- Fitness and Coordination: *Increasing integration of body parts through varied movement experiences.*
- Connection and Communication with others: *Developing social attunement to others through shared dance and movement experiences*

For more information:
If you would like to learn more about the dance movement therapy program, please feel free to contact Tessa Hens (on email xxx or after the session), who can provide readings and website links for further information.

Assessment: Dance movement therapist's observations

Objective 1. Fitness and coordination, increasing integration of body parts through varied movement experiences:

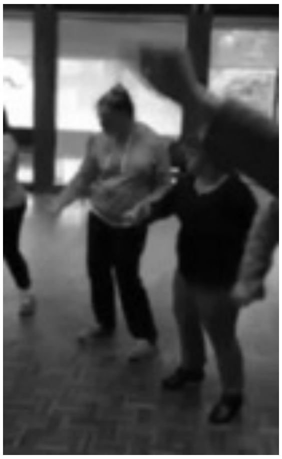

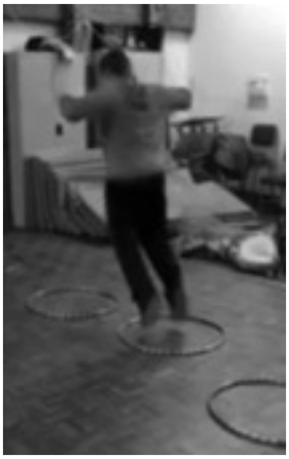

Angela's confidence to participate in group routines and to experiment with new movement experiences is slowly growing as she settles into the group. She has a preference for light, small movements using her hands, arms and core. This term we have been experimenting with integrating body parts to move in and through space. For example, the group used a range of props to create their own dance obstacle courses which they moved through to different styles of music. Angela was able to demonstrate rhythmic walking, using high and low level movements and jumping (see film attached). She has also become more confident in sharing movement during our group greeting of improvised movement, and has demonstrated bi-lateral patterning with a strong dynamic using hand punches

At the end of each session, cool down movements include a spinal roll (graduated forward roll through the spine), core twists, breath work and stretching. Initially Angela preferred to just dip her head and upper body forward. With gentle guidance through touch and mirrored modelling, Angela has achieved a deeper and more fluid forward fold, demonstrating the ability to extend her movements and integrate body parts more fully with support.

Objective 2. Connection and communication with others, developing social attunement to others through shared dance and movement experiences:

Angela is a quieter member of our group but has demonstrated the ability to take turns, listen to others, and offer her own news during group greeting, and contribute movement ideas to group warm ups. Her voice volume and confidence when interacting has begun to grow in the second half of this series of sessions. She has benefited from the support of her closer friends in the group who have helped her to settle into group routines with guidance and encouragement.

During partner and small group work, Angela demonstrates warm and sensitive connections with others using eye contact and gesture to check in with her peers. She has also worked in small teams effectively by offering ideas and listening to others to generate movement ideas and shapes. Angela has demonstrated an emerging ability to mirror movement with peers she feels comfortable with and with staff members. Angela is always calm and friendly in her interactions and her smiles indicate enjoyment of working in a supportive group setting.

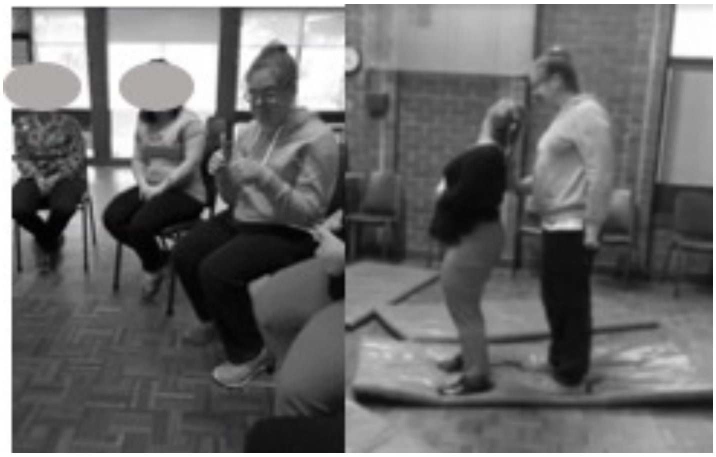

Future areas of focus and development

The group dynamic established in our sessions is intended to be supportive and encouraging. Peers often support and encourage each other in their learning and expressive movement. This assists Angela to gain confidence in connecting with others and to experience enjoyable interactions that utilise both verbal and non-verbal communication. In this setting she can rely on her body as much as her voice to connect with others, creating more openings for social success and confidence building. Angela may benefit from extending on her social interactions in this group by continuing to work with this group of peers with whom she has built positive rapport.

This warm group dynamic is intended to supportsAngela to feel safe in experimenting with new movement and experiences. In future Angela may benefit from experimenting with a more diverse range of movement dynamics. Areas of possible extension include movements using ‘strong’ weight and grounded postures, free flow sequences that allow her to release more into movement and increase extension in her limbs.

Photos and video clips:

Three short video clips of Angela participating in dance movement therapy are attached with this report. They show:

1. Angela contributing a ‘punch’ movement for the group to follow, during our circle warm up using strong rhythmic movement;
2. Angela working with a partner to bring awareness to spines through touch. Both partners took turns to trace the outline of the spine on each other’s bodies. Angela indicated that she enjoyed working with her partner, smiling and leaning towards her to speak;
3. Angela jumping and running in a dance obstacle course created by the group.

Assessment: Participant's response

Present: Angela and Tess Hens. **Evaluation Date**: 19 June, 2017

Dance program goals

This term we have been working on:

1. Getting on with others by dancing together.

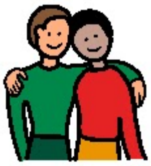

1. Getting better at using our different body parts together and on their own.

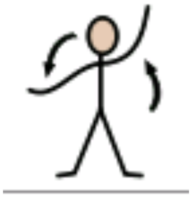

Individual photos and film: Watch, respond and discuss:

Tess and Angela looked at and then discussed the dance program objectives. Angela affirmed her agreement with them. Then Tess and Angela viewed short videos and photos of her dancing, relaxing and interacting with her peers during dance sessions. Tess invited Angela to comment by asking questions:

T: 'Angela can you tell me what you think we have been learning about in the dance program?'
A: 'Movement, dancing', 'I learned about my spine.'

T: 'What do you enjoy?'
A: 'Hand movements; relaxation'
'I was having fun watching myself. I liked looking at pictures of myself.
Good to see myself happy. That feels good. I'm enjoying it.'
